# Complement Genome Annotation Lift Over Using a Weighted Sequence Alignment Strategy

**DOI:** 10.3389/fgene.2019.01046

**Published:** 2019-11-13

**Authors:** Baoxing Song, Qing Sang, Hai Wang, Huimin Pei, XiangChao Gan, Fen Wang

**Affiliations:** ^1^The Department of Life Science, Qiannan Normal College for Nationalities, Duyun, China; ^2^Department of Comparative Development and Genetics, Max Planck Institute for Plant Breeding Research, Köln, Germany; ^3^Institute for Genomic Diversity, Cornell University, Ithaca, NY, United States; ^4^Department of Plant Developmental Biology, Max Planck Institute for Plant Breeding Research, Köln, Germany; ^5^Biotechnology Research Institute, Chinese Academy of Agricultural Sciences, Beijing, China

**Keywords:** weighted sequence alignment, genome annotation, genome-wide multiple-sequence alignment, genetic variants uniformization, gene expression level quantification

## Abstract

With the broad application of high-throughput sequencing, more whole-genome resequencing data and *de novo* assemblies of natural populations are becoming available. For a particular species, in general, only the reference genome is well established and annotated. Computational tools based on sequence alignment have been developed to investigate the gene models of individuals belonging to the same or closely related species. During this process, inconsistent alignment often obscures genome annotation lift over and leads to improper functional impact prediction for a genomic variant, especially in plant species. Here, we proposed the zebraic striped dynamic programming algorithm, which provides different weights to genetic features to refine genome annotation lift over. Testing of our zebraic striped dynamic programming algorithm on both plant and animal genomic data showed complementation to standard sequence approach for highly diverse individuals. Using the lift over genome annotation as anchors, a base-pair resolution genome-wide sequence alignment and variant calling pipeline for *de novo* assembly has been implemented in the GEAN software. GEAN could be used to compare haplotype diversity, refine the genetic variant functional annotation, annotate *de novo* assembly genome sequence, detect homologous syntenic blocks, improve the quantification of gene expression levels using RNA-seq data, and unify genomic variants for population genetic analysis. We expect that GEAN will be a standard tool for the coming of age of *de novo* assembly population genetics.

## Introduction

For large-scale population genetics projects, a high-quality reference genome sequence is generally assembled, and great efforts are exerted to generate a high-quality reference genome annotation. The genotypic data of a group of taxa are compared with the reference genome sequence to uncover genotypic variants. For whole-genome resequencing projects, single-nucleotide polymorphisms (SNPs), insertions and deletions (INDELs), and other complex variants were called via short reads mapping (Danecek et al., 2011; Gan et al., 2011). The decreasing costs and advancements of *de novo* genome sequencing and assembly have revolutionized the ability to investigate sequence diversity (Xiao et al., 2017; Ruan and Li, 2019), thereby allowing international consortiums of geneticists and researchers to develop foundational resources for human, animal, and plant genomes (Consortium, 2015; Alonso-Blanco et al., 2016; Wang et al., 2018; Ruan and Li, 2019).

With the growing quantity of genomic information available, the ability to integrate information from different species and/or accessions becomes an interesting challenge. One of the crucial steps is to “lift over” the reference coordinates to another genome sequence (Hinrichs et al., 2006; Spudich and Fernández-Suárez, 2010; Swain et al., 2012; Zhao et al., 2014). For whole-genome resequencing data, the pseudo-genome sequence can be obtained by replacing the reference alleles with alternative alleles. The lift over of the reference genomic coordinates to the pseudo-genome sequences (Zhao et al., 2014) can be performed by counting the number of base pairs that have been shifted by the upstream variants (Wang et al., 2010; MacArthur et al., 2012). For *de novo* assembly genomes, the reference coordinates can be lifted with the genome sequence alignment-based variants calling.

**Figure 1:**
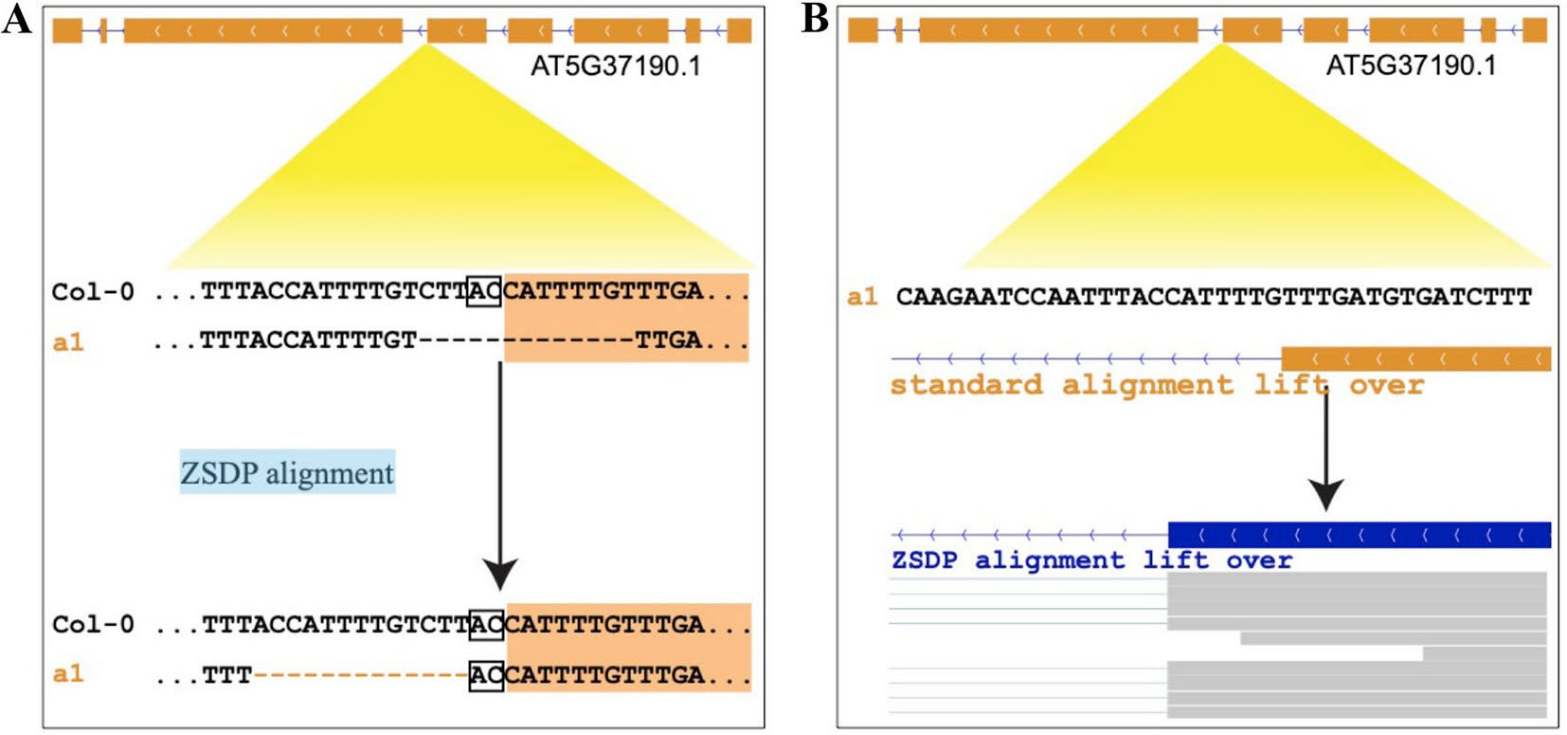
Example of inconsistent sequence alignments that affect variant functional inference. **(A)** Upper panel standard sequence alignment suggests that a 13-bp deletion disturbs the splice site and shifts the ORF of AT5G37190.1. Lower alignment panel suggests that a 13-bp deletion is located in the intron, and the splice site is conserved. **(B)** Upper panel shows the annotation of AT5G37190.1 of *a1* allele by coordinate lift over using standard sequence alignment. Lower panel shows the annotation updated by our ZSDP algorithm, and the RNA-seq reads mapping support the ZSDP result.

As previous studies point out, the same variants, especially INDELs and nearby SNPs, might be represented in multiple ways. The left alignment and genome-wide multiple-sequence alignment (MSA) have been proposed to solve this problem in population genetics studies (Tan et al., 2015; Song et al., 2018). While the ambiguity of variant representation could cause false-positive open-reading-frame (ORF) state interruption prediction (**Figure 1**), there are very few well-designed pipelines that can perform genome annotation lift over and take INDEL inconsistent alignment into consideration. Thus, the generation of false-positive ORF-shift predication and improper genotypic variant functional prediction is expected and has been observed.

Here, we provide a semi-global sequence alignment algorithm to refine genome annotation lift over by giving distinct weights to different genetic regions. We named this weighted-sequence alignment approach as zebraic dynamic programming (ZDP). ZDP is accelerated by extending the striped Smith–Waterman (SSW) algorithm (Farrar, 2007); we named the speed up version as zebraic striped dynamic programming (ZSDP). The ZSDP has been implemented in our open-source software GEAN. In the subsequent sections, we demonstrate the performance of ZSDP by aligning *Arabidopsis thaliana*, *Cardamine hirsuta* (Gan et al., 2016), *Drosophila melanogaster*, and maize (*Zea mays*) genome sequences. First, by using the *A. thaliana* and *D. melanogaster* genomes, we develop concepts that underlie the algorithms of GEAN as a tool to project a reference genome annotation to a new genome sequence assembly of the same species and solves inconsistent alignment problems. We then consider complex crop genomes and inter-species genomes and try to transform the genome annotation of the inbred maize line B73 to Mo17 and *A. thaliana* to *C. hirsuta*. We also show that the lift over genome annotation can help perform variant calling for the *de novo* assembly genome sequence and genome-wide MSA. As far as we know, GEAN is the first implementation that has been designed to handle the inconsistent alignment problem for gene structure annotation lift over.

## Result

### Mitigation of False Deleterious Variants Caused by Inconsistent Alignment

An INDEL/SNP ratio of ∼25% has been observed in plants, animals, and humans (Rakocevic et al., 2019). At least 63–65% of INDELs, as well as nearby SNPs, might be affected by inconsistent alignments in a population (Song et al., 2018). As shown in **Figure 1A**, the standard sequence alignment-based annotation projection could lead to false loss-of-function prediction in genic regions and improper predictions of genetic load in variants. Genetic load of genotyped variants has been analyzed for various purposes by using the data from whole-genome resequencing projects, e.g., GWAS burden test (Song et al., 2018), deleterious mutations (Ramu et al., 2017), and synonymous–nonsynonymous mutation rates for natural selection analysis (Nekrutenko et al., 2002; Liu et al., 2008). Here, we show the scale of transcripts, without variants, shifting the ORF and disturbing splicing sites that could be falsely predicted as loss of function.

**Figure 2:**
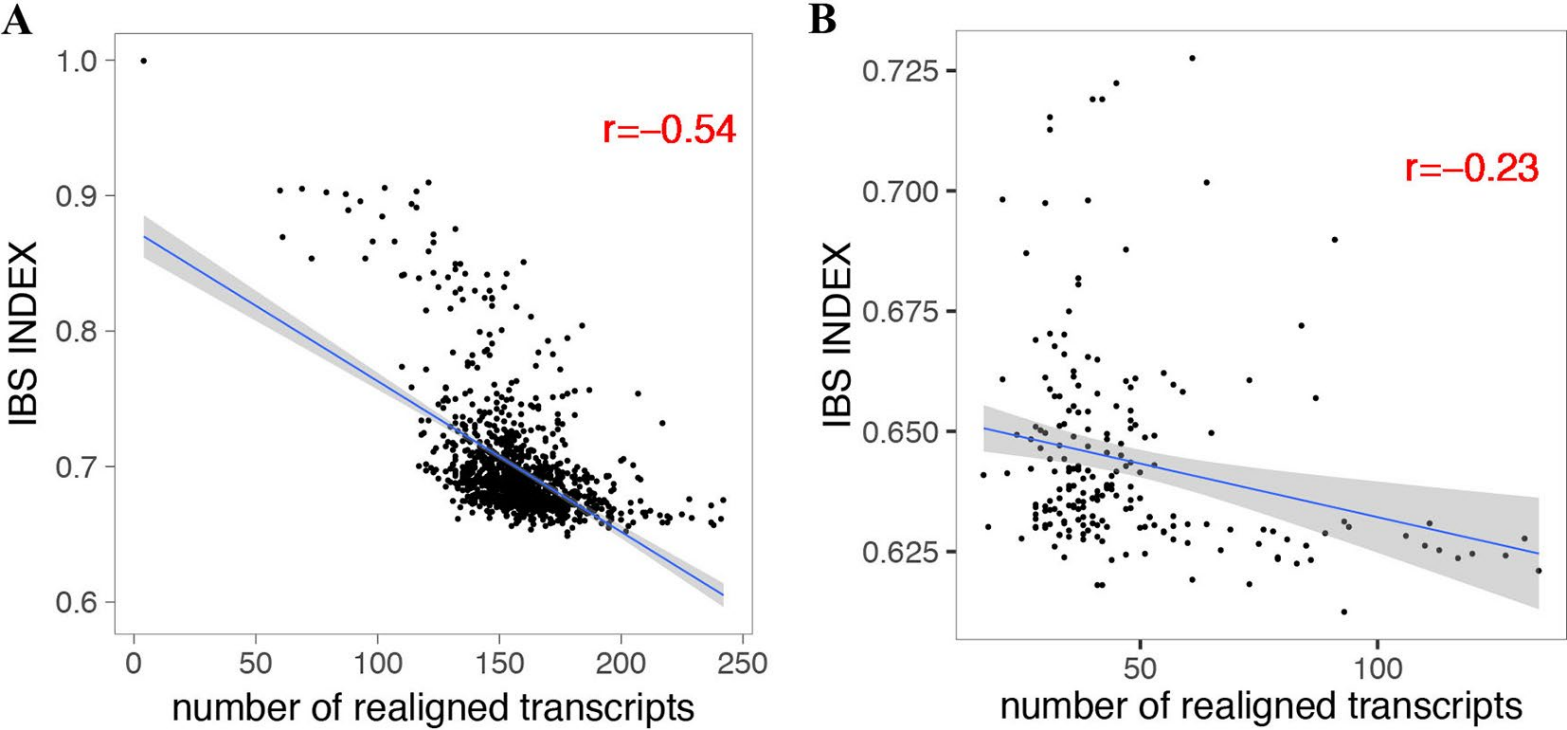
Correlation between the number of realigned transcripts by the ZSDP method and identical by state index for *A. thaliana* **(A)** and *D. melanogaster* **(B)**.

By using the variant calling results of 1,211 *A. thaliana* accessions (Alonso-Blanco et al., 2016) and 203 *D. melanogaster* lines (Huang et al., 2014; Dembeck et al., 2015) using IMR/DENOM (Gan et al., 2011), we created the pseudo-genome sequence of each accession and performed standard genome annotation lift over. For the lifted protein-coding transcripts that were predicted as loss of function, we realigned the gene annotation by using the ZSDP algorithm. ZSDP reannotated a total of 6,713 transcripts as ORF state conserved for *A. thaliana* accessions and 1,946 transcripts for *D. melanogaster* population. An average of 158 and 47 transcripts were realigned as ORF state conserved for each *A. thaliana* and *D. melanogaster* accession, respectively. We observed negative correlations (p < 2.2e-16) between the number of realigned transcripts and identical by state (IBS) index for both species (**Figure 2**), which suggested that more transcripts have been affected by inconsistent alignment between individuals with increased diversity.

To evaluate the result generated by the ZSDP approach against standard sequence alignments, we compared the genome annotation files with RNA-seq results using Cufflinks v2.2.1 (Trapnell et al., 2012). Raw RNA-seq reads from 728 *A. thaliana* accessions released by the Arabidopsis 1001 Epigenomes project (Kawakatsu et al., 2016) were trimmed with Trimmomatic (Bolger et al., 2014) (v0.36, LEADING:10 TRAILING:10 SLIDINGWINDOW:4:15 MINLEN:6) and mapped to pseudo-genome sequence using Hisat2 (Kim et al., 2015) (v2.1.0, --max-intronlen 30000). After filtering low-quality accessions (less than 3,000,000 RNA-seq reads available, alignment rate ≤90%), 697 accessions were left. The generated bam files were fed into Cufflinks v2.2.1 to get all splice site positions. For controversial splice sites between standard coordinate lift over and ZSDP, 41,235 controversial split sites could not be determined by the RNA-seq reads due to absence or low expression levels of the realigned transcript isoform, while the RNA-seq data confirmed 23,448 ZSDP splice sites, which is ∼20 times that of the RNA-seq confirming pre-updated lift over of splice sites (1,200).

For regions with potential false loss-of-function variant records, GEAN realigned the transcripts, recalled the variants, and replaced the old variants with the realigned variant records. The newly realigned variant records could be used to predict the functional annotation of genetic variants (Wang et al., 2010), etc.

### Non-Reference Line RNA-seq Reads Mapping Rate Could Be Improved Using Pseudo-Genome Sequence

For many non-reference accession RNA-seq projects, the reference genome sequence is usually used for RNA-seq read mapping (Kawakatsu et al., 2016; Krizek et al., 2016). Here, we tested whether mapping reads to the pseudo-genome sequence could generate an increased mapping rate and expression level quantification for any particular gene. For this hypothesis, we set up a pipeline to generate a comprehensive genome annotation of pseudo-genome sequence. Using the GFF file from coordinate lift over, the ORF states of the target line could be checked with the inferenced haplotype sequence (Xiang et al., 2016). Any ORF state disruption, according to the lift over results, was realigned with the ZSDP algorithm.

**Figure 3:**
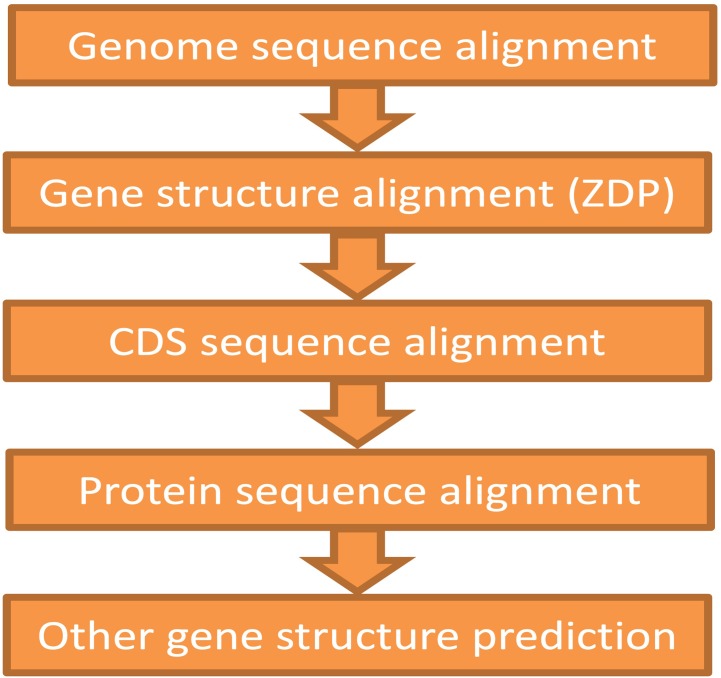
Schematic showing the pipeline for the protein-coding gene annotation of the non-reference genome. The annotation of upstream function would be supplemented with the downstream modules.

The ZSDP algorithm could migrate the reference genome annotation to the pseudo-genome sequence. If the new accession contained variations in gene structure (i.e., number of exons that changed, alternative splice site, novel genes), GEAN complemented the annotation lift over with orthologue-based annotation (Slater and Birney, 2005), the results of an external *ab initio* annotation method (Stanke and Waack, 2003), and transcript assembly (Trapnell et al., 2012). As illustrated in **Figure 3**, the gene structure predicted by the higher module would first be adapted, and the region predicted as ORF state shift or noncoding would be handled by the below modules. On average, 346 transcripts were re-annotated using ZSDP or Exonerate (Slater and Birney, 2005) for each accession.

**Figure 4:**
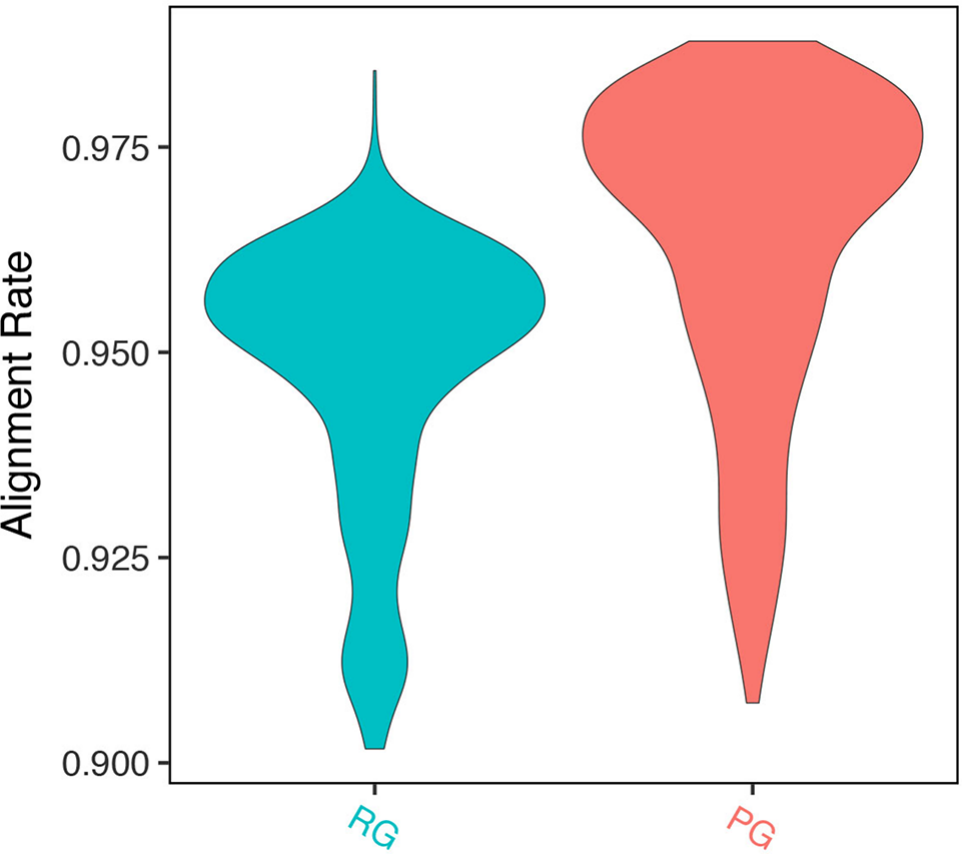
Alignment rate of RNA-seq read to pseudo-genome (PG) sequence and Col-0 reference genome (RG) sequence.

We mapped RNA-seq reads of 728 *A. thaliana* accessions (Kawakatsu et al., 2016) to the Col-0 reference genome sequence and pseudo-genome sequence separately. The genome annotations for each pseudo-genome were obtained using the genome annotation pipeline in GEAN. *Ab initio* annotation predictions from Augustus (Stanke and Waack, 2003) (--genemodel = complete --maxDNAPieceSize = 2000000 --sample = 200) were used as the fifth modules in our pipeline. Raw RNA-seq reads were trimmed and mapped as described earlier. Mapped reads were counted using HTSeq 0.9.1 (Anders et al., 2015) with default parameters. The mapping rate to the pseudo-genome sequence was significantly higher than that to Col-0 reference genome sequence (94.76% vs. 96.33%, p value < 2.2e-16, Wilcoxon signed rank test) (**Figure 4**).

**Figure 5:**
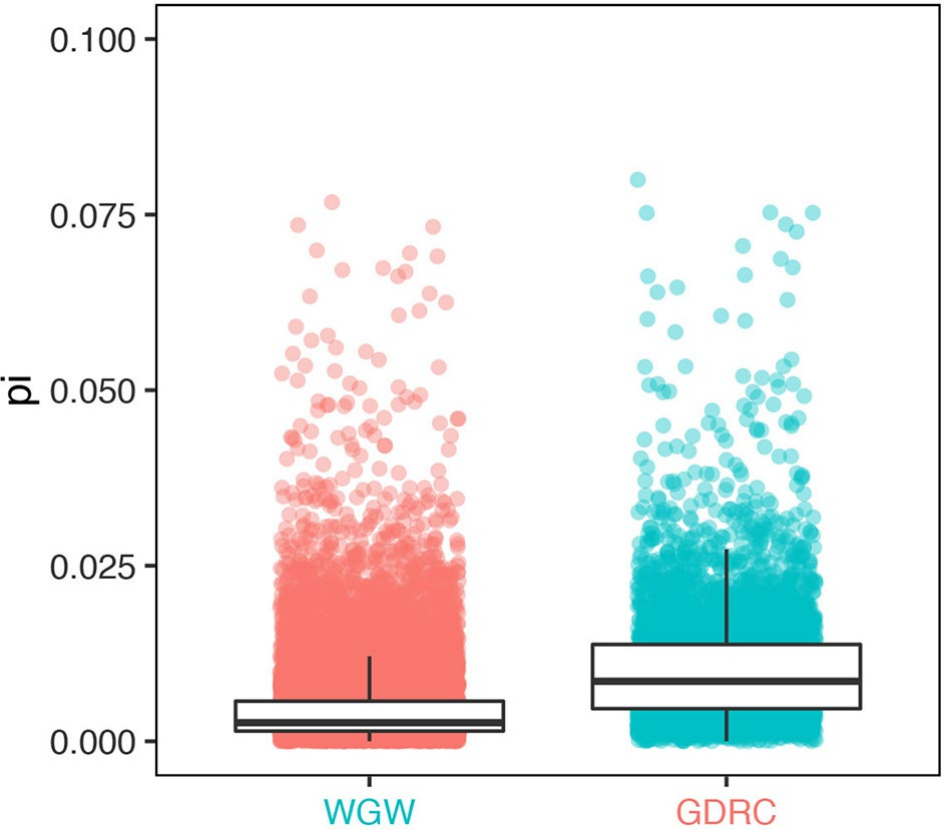
Sequence diversity of genes with significantly different read counts (GDRC) versus the whole-genome-wide (WGW) background.

Due to the improved mapping rate, a group of 5,122 genes had significantly higher expression levels when quantified by pseudo-genome structure annotation (significantly higher is roughly defined as larger than Q3+5* IQ, Q1 is the 25^th^ percentile, Q3 is the 75^th^ percentile, and IQ = Q3−Q1. detected with R command boxplot.stats(log(reads_count)ratio, coef = 5)$count). Sequence diversity (measured using pi value) of these genes was significantly higher than that of the genome-wide background (Wilcoxon test p value < 2.2e-16). Gene ontology (GO) analysis was conducted using agriGO (Du et al., 2010) with default settings; the results suggested that using the GEAN pipeline was essential for gene expression level quantification in more diverse genes (**Figure 5**), especially genes with GO terms related to cell death, defense response, and immune response (**Supplementary Figure 1**).

### Lift Over Reference Genome Annotation to *De Novo* Assembly Genome Sequence

We implemented a genome annotation lift over function in GEAN by utilizing the genome sequence alignment information, e.g., generated by MUMmer 4, minimap2 (Li, 2018; Marçais et al., 2018). The genome alignment results typically provide a pair of ranges: one is from the reference sequence and another from its corresponding range in the query genome sequence. GEAN performs pairwise sequence alignment with a very efficient sliding window method. It lifts the reference genome annotation to the query genome sequence and updates the annotation using the ZSDP algorithm.

**Figure 6:**
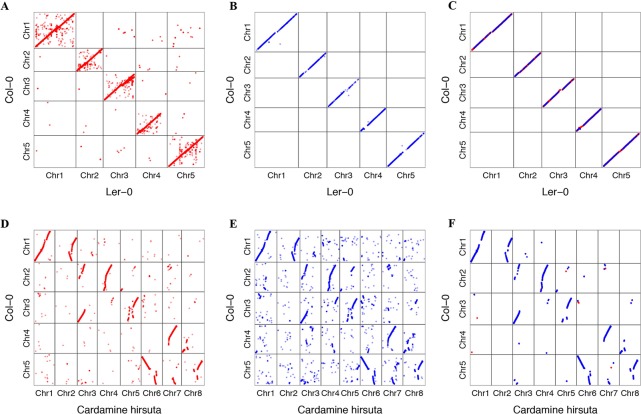
Dot plot of gene position in Col-0 against the position of genes transformed to other *de novo* assemblies. Dots in red are the result of standard sequence alignment lift over, and those in blue have been realigned by the ZSDP algorithm. **(A)** Project *Arabidopsis thaliana* Col-0 genome annotation to the L*er*-0 accession genome sequence using standard sequence alignment. **(B)** Project Col-0 genome annotation to L*er*-0 genome sequence of those genes could not be transformed using standard sequence alignment using ZSDP algorithm. **(C)** Highlighting the syntenic blocks between Col-0 and L*er*-0 using the transformed annotations. **(D)** Project *A. thaliana* Col-0 annotation to the *Cardamine hirsuta* genome sequence using standard sequence alignment. **(E)** Project Col-0 annotation to *C. hirsuta* genome sequence of those genes could not be transformed using standard sequence alignment using ZSDP algorithm. **(F)** Highlighting the syntenic blocks between *A. thaliana* Col-0 and *C. hirsuta* using the transformed annotations.

We tried to transform the genome annotation of *A. thaliana* Col-0 to *A. thaliana* L*er*-0 and *C. hirsuta* genome sequence. *A. thaliana* L*er*-0 was assembled into the chromosome level (Zapata et al., 2016). *C. hirsuta* is closely related to *A. thaliana* phylogenetically, whose genome has previously been published (Gan et al., 2016). We found that 34,869 protein-coding transcripts of *A. thaliana* Col-0 accession could be transformed to L*er*-0. A total of 32,307 transformed annotations had a conserved ORF state, and 752 of those annotations were realigned by the ZSDP algorithm. A total of 14,905 Col-0 protein-coding transcripts could be transformed to *C. hirsute* as having a conserved ORF state, while 7,119 of these were realigned by the ZSDP algorithm. The higher proportion of transcripts realigned by ZSDP in *C. hirsute* confirms the negative correlations observed between the number of realigned transcripts and the identical by state index. A dot plot of the transformed gene annotation from Col-0 to *C. hirsute* was comparable with the Circos plot that shows the synteny of *A. thaliana* and *C. hirsute* genomes published before (Gan et al., 2016). In addition, a larger proportion of genes that did not follow the large synteny fragments were observed in *C. hirsute* than in L*er*-0 (**Figure 6**). Maize is a widely researched crop species and has one of the most complex and diverse genomes (Jiao et al., 2017). We used GEAN to transform Maize B73 AGPv3 genome annotation to one of the high-quality genome assembly Mo17 (Yang et al., 2017). A total of 43,564 out of 63,780 protein-coding transcripts could be lifted over as ORF state conserved, and 5,101 protein-coding transcripts were realigned by ZSDP. The proportion of ZSDP realigned transcripts is higher than Col-0 to L*er*-0 while lower than lifting *A. thaliana* to *C. hirsute*.

### Base-Pair Resolution Variant Calling for *De Novo* Assembly Genome Sequence

Variant calling is important for various population genomics and genetics analyses. We implemented a pipeline in GEAN to perform genome-wide sequence alignment and variant calling using the lift over annotations as anchors. In detail, we aligned the annotation of *de novo* assembly genome sequence with the reference annotation using a Needleman–Wunsch algorithm and treating identical gene IDs as a match. The start and stop codons of matched genes were used as anchors to split the whole-genome sequence into fragments, and a sliding window method was used to perform base-pair resolution sequence alignment and variant calling in each fragment. By applying this method on a L*er*-0 *de novo* assembly genome sequence (Zapata et al., 2016), 2,664,186 SNPs and 546,778 INDELs were detected, which is higher than those from whole-genome resequencing variant calling (693,834 SNPs and 159,350 INDELs) (Song et al., 2018). The larger number of variants could contribute to the high power of variant calling of *de novo* assembly. GEAN decomposes structural variation, e.g., relocation, into SNP or INDEL, and thus increases the number of variants. Interestingly, the INDEL/SNP ratios for the two results are comparable with each other. We aligned the Col-0 and L*er-*0 genome sequence using MUMmer4 (--maxmatch -c 100 -b 500 -l 50) and performed structural variant calling with Assemblytics (Nattestad and Schatz, 2016; Marçais et al., 2018) and detected 3,642 variants >50bp, which is less than that of GEAN (6380). This difference may be due to the MUMmer4+Assemblytics pipeline generating fragmented alignments and, thus, could not align every base pair in the genome (**Supplementary Figure 2**). Under the GEAN pipeline framework, all the variants will be recorded. Given a reference genome sequence, GEAN could infer the *de novo* assembly genome with the variant calling result. As far as we know, GEAN is the first software that can call variants in such a comprehensive manner.

### Genome-Wide Multiple-Sequence Alignment

For the variant calling of a population of individuals, inconsistent alignments could generate inconsistent variant records. To unify variants, left alignment approach and genome-wide MSA have been developed (Tan et al., 2015; Song et al., 2018) while neither left alignment method nor previous genome-wide MSA counting the gene structure.

To conserve genome annotation lift over, GEAN performs MSA for each genetic feature separately. To make alignments faster, GEAN tries to use short and nonoverlapping fragments to run the MSA algorithm. Considering that the boundary of genetic features of functionally conserved transcripts could be well defined, GEAN performs MSA for short elements [e.g., intron, coding DNA sequence (CDS), intergenic region] directly and only uses an overlapping sliding window to perform MSA only for elements longer than sliding window size, which is a configurable parameter.

## Method

### Definitions

The ORF state has been defined by updating the previous rules (Song et al., 2018) as follows: 1) Splicing sites is one motif in a “SpliceSites” file, which is included in the released software; 2) The minimum intron length is larger than a threshold value; 3) CDS sequence length is larger than a parameter; 4) The length of CDS sequence is divisible by 3; 5) There is no premature stop codon; 6) The sequence ends with a stop codon; 7) The sequence starts with a start codon. The input DNA sequence ambiguity nucleic acid encoded using International Union of Pure and Applied Chemistry could be well dealt with.

### Coordination Lift Over

Considering the presence of INDELs, SVs, and sequence fragment rearrangements, the coordinates of certain orthologous haplotype sequence fragments are different for each individual. Lift over is a way of mapping reference coordinates from one genome assembly to another.

For whole-genome resequencing projects, GEAN re-implemented the approach published previously (Gan et al., 2011) and performs lift over by counting the number of base pairs that have been shifted by the upstream variants. For the *de novo* assembly sequence, considering the similar range entries as input [e.g., Mummer (Marçais et al., 2018), minimap2 (Li, 2018, 2)], GEAN generates the base-pair resolution pairwise sequence alignment with a dynamic programming algorithm by using a sliding window, which is used in the coordinate lift over.

### Zebraic Dynamic Programming Algorithm

We designed a pairwise sequence alignment algorithm taking gene structure into consideration by extending the standard sequence alignment dynamic programming algorithms (Needleman and Wunsch, 1970; Smith and Waterman, 1981; Gotoh, 1982). The two sequences to be compared, query sequence and reference sequence, are defined as Q = q_1_, q_2_ … q_m_ and D = d_1_, d_2_ … d_2_, respectively. The length of the query and reference sequences are m = |Q| and n = |D|, respectively. A substitution matrix W(q_i_, d_j_) is defined for all residue pairs. The score W(q_i_, d_j_) ≤ 0 when q_i_ ≠ d_j_, and W(q_i_, d_j_) > 0 when q_i_ = d_j_. The penalty for opening and extending a gap is defined as G_open_ and G_ext_, respectively.

**Figure 7:**
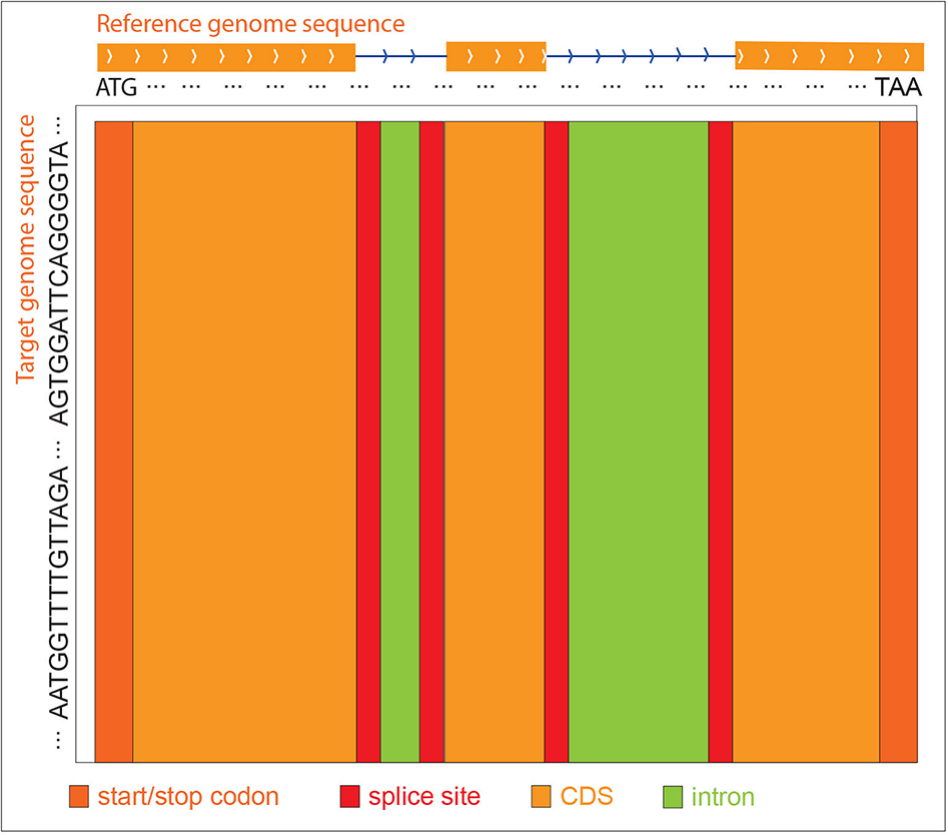
ZDP sequence alignment methods. When the reference genome sequence is aligned to the target accession genome sequence, different scoring strategies are used for distinct reference regions to construct the score matrix. The purpose is to align the exon regions preferentially.

Since the gene structure of the reference sequence, i.e., intron, CDS, start codon, stop codon, and splice sites are known, GEAN uses the reference sequence from the start codon to the stop codon for alignment. The query sequence is, in general, extended both upstream and downstream by the length of the gene to make sure the genetic region is included in the selected region. When constructing the score matrix, we initialize the first row and the first column with 0. At the traceback step, GEAN starts the tracing from the cell with the largest value in the last column, since we expect that the reference sequence could be globally aligned, while the query sequence could be locally aligned. Different W(q_i_, d_j_), G_open_, and G_ext_ values were used for intron, CDS, splice sites, and start and stop codons separately, so we have all the score strategies as W(q_i_, d_j_)_intron_, G_open_intron_, G_ext_intron_, W(q_i_, d_j_)_CDS_, G_open_CDS_, G_ext_CDS_, W(q_i_, d_j_)_spliceSites_, G_open_spliceSites_, G_ext_spliceSites_, W(q_i_, d_j_)_start/stopCodon_, G_open_start/stopCodon_, and G_ext_start/stopCodon_.The protein-coding region and splice sites will be highly weighted, and the sequence in those regions will be aligned primarily (**Figure 7**). Thus, any ambiguity in ORF states caused by different genetic variance representation could be polished to keep the ORF states complete.

As we need to determine which gene structure elements of the current j are located and to avoid repeating this query process for traceback, we defined a traceback matrix T with the same size as the score matrix, which records the scoring path of each cell of the score matrix. The value of each cell in the traceback matrix is one of SNP, INSERTION, DELETION, or an ambiguous value SNPORINSERTION, SNPORDELETION, INSERTIONORDELETION, SNPORINSERTIONORDELETION.

The alignment scores ending with a gap along D and Q are E calculated using Equation (1) and F calculated using Equation (2), respectively:

(1) $$E_{i,j}=\left\{\begin{array}{ccc} E_{i,j-1}-G_{\mathrm{ext}} & if\;(T_{i,j-1}\;\mathrm{is}\;\mathrm{INSERTION}\;\mathrm{or}\;\mathrm{SNPORINSERTION}\;\mathrm{or}\; & \\ & \begin{array}{l} \mathrm{SNPORINSERTION}\;\mathrm{or}\\ \mathrm{INSERTIONORDELETION}) \end{array} & \\ H_{i,j-1}-G_{\mathrm{open}} & & \mathrm{otherwise} \end{array}\right\}$$

(2) $$F_{i,j}=\left\{\begin{array}{ccc} E_{i-1,j}-G_{\mathrm{ext}} & \mathrm{if}\;(T_{i,j-1}\;\mathrm{is}\;\mathrm{INSERTION}\;\mathrm{or}\;\mathrm{SNPORINSERTION}\;\mathrm{or}\; & \\ & \begin{array}{l} \mathrm{SNPORINSERTION}\;\mathrm{or}\\ \mathrm{INSERTIONORDELETION}) \end{array} & \\ H_{i-1,j}-G_{\mathrm{open}} & & \mathrm{otherwise} \end{array}\right\}$$

(3) $$M_{i,j}=M_{i-1,j-i}+W(q_{i},d_{j})$$

The alignment score for H_i,j_ where 1≤i≤m and 1≤j≤n is defined by Equation (4):

(4) $$H_{i,j}=\max\left\{\begin{array}{c} E_{i,j}\\ F_{i,j}\\ M_{i,j} \end{array}\right\}$$

The matrix T was updated as:

(5) $$T_{i,j}=\left\{\begin{array}{cc} \mathrm{MATCH} & if\;M_{i,j}>E_{i,j}\;\mathrm{and}\;M_{i,j}>F_{i,j}\\ \mathrm{DELETION}, & if\;F_{i,j}>E_{i,j}\;\mathrm{and}\;F_{i,j}>M_{i,j}\\ \mathrm{INSERTATION}, & if\;E_{i,j}>F_{i,j}\;\mathrm{and}\;E_{i,j}>M_{i,j}\\ \mathrm{SNPORDELETION}, & if\;F_{i,j}>E_{i,j}\;\mathrm{and}\;F_{i,j}==M_{i,j}\\ \mathrm{SNPORINSERTION}, & if\;E_{i,j}>F_{i,j}\;\mathrm{and}\;M_{i,j}==M_{i,j}\\ \mathrm{INSERTIONORFDELETION}, & if\;E_{i,j}==F_{i,j}\;\mathrm{and}\;E_{i,j}>M_{i,j}\\ \mathrm{SNPORINSERTIONORDELETION} & if\;E_{i,j}==F_{i,j}\;\mathrm{and}\;E_{i,j}==M_{i,j} \end{array}\right\}$$

Once *T_ij_* is set as a certain value, i.e., SNP, INSERTION, DELETION, all the continuous ambiguous ancestral cells will be set as a certain value, until a certain value is found.

For RAM-saving purposes, instead of keeping the whole H_i,j_ matrix, ZDP only keeps the last row and last column, and the traceback step can be easily performed using the traceback matrix.

### Zebraic Striped Dynamic Programming for Genome Annotation Alignment

ZSDP is a faster version of the ZDP algorithm and generates identical results. The acceleration in speed was achieved by intra-sequence parallelization, which parallelizes the algorithm by extending the SSW algorithm (Farrar, 2007) to a semi-global sequence alignment. The SSW algorithm could be 10 times faster than the standard Smith–Waterman algorithm using AVX2 and has been widely embedded in several high-throughput sequencing read mapping software, such as Bowtie2 (Langmead and Salzberg, 2012, 2), BWA-SW (Li and Durbin, 2010), Stampy (Lunter and Goodson, 2011), and minimap2 (Li, 2018).

Under the SSW algorithm framework, when calculating H_i,j_, the value from the scoring matrix W(q_i_, d_j_) is added to H_i-1,j-1_. To avoid finding W(q_i,j_) for each cell, a query profile parallel to the query is calculated for each possible residue. The query profile is calculated once for each alignment. The calculation of H_i,j_ only requires the addition of the pre-calculated score to H_i-1,j-1_. The ZSDP method takes a similar approach by pre-calculating a query profile for each gene structure element category separately and uses a standard method to implement the lazy F evaluation loop.

The SSW method only provides the optimal alignment score but does not report the information necessary to construct the final alignment. The SSW Library reports the detailed alignment by performing SSW twice (get the ending positions by a forward SSW and then generates the beginning position by a backward SSW) and performs a standard Smith–Waterman alignment by aligning the sequences between the beginning and ending positions (Zhao et al., 2013), which should be further optimized.

Here, we constructed the H_i,j_ vector by comparing H_i-1,j-1_ and E_i,j_ and stored the H_i,j_ from vHstore into the standard integer scoring matrix. We calculated the F_i,j_ score for each cell of the current column. By comparing the F_i,j_ value with the H_i,j_ value, we updated the H_i,j_ and vHstore with the larger value. The corresponding value in the traceback matrix was also updated. Then, the traceback step of the dynamic programming algorithm, which provides the detailed alignment, could be performed using the traceback matrix.

The improvements in computational time is related to the number of cells calculated per central processing unit register. GEAN uses 8, 16, and 32 bits to process the score matrix for sequence with different lengths and puts as many cells as possible into every single instruction multiple data register. Meanwhile, the integer type is wide enough to process the sequence alignment scores. We implemented the striped dynamic algorithm with AVX2. AVX2 is available for most modern Intel processors, whose register is 256 bits wide.

### Sequence Alignment by Sliding Window

**Figure 8:**
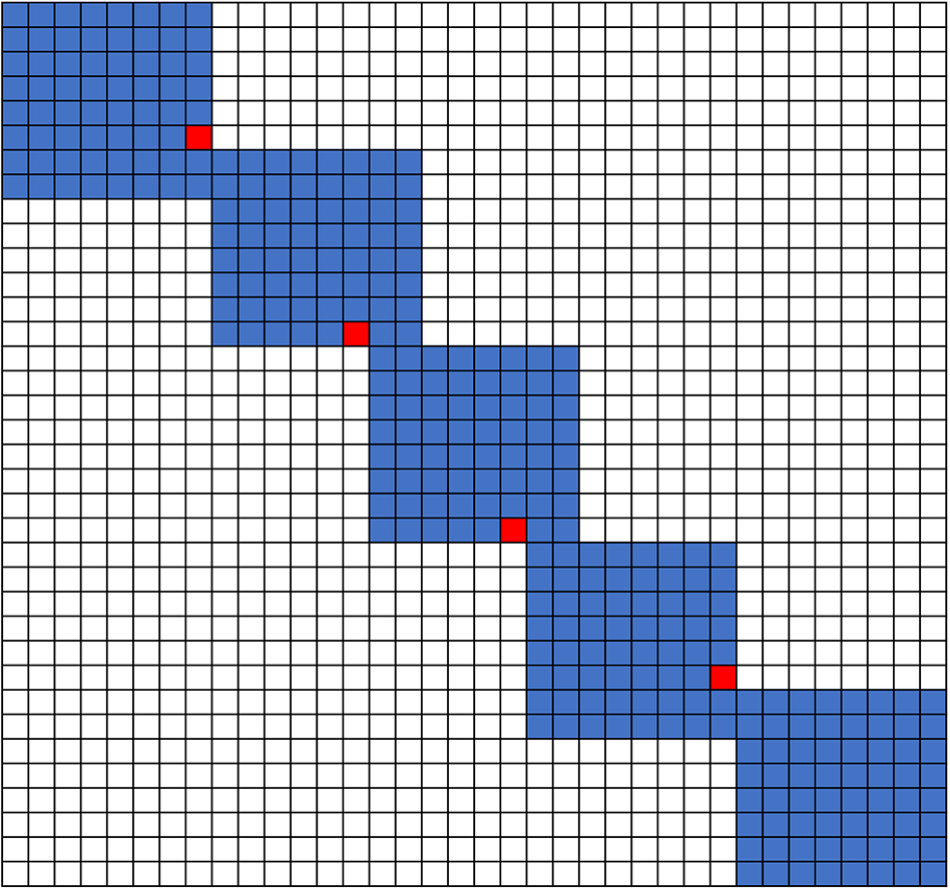
Sliding window sequence alignment method used for the long sequence alignment.

To accelerate the alignment of long sequences, we used a sliding window method. For each window, we determined the maximum value of the last row or column. The maximum cell would be used as the start cell of the next scoring window (**Figure 8**). With this strategy, we have a linear computational time cost. Similar to banded alignment, we note that this type of heuristic can fail to provide the optimal alignment under some situations. Luckily, the failures in these alignments are always only present in small local regions. A large sliding window size could be used to avoid this problem, which is a configurable parameter from the command line. This problem could be avoided, if the sliding window is larger than the sequence being aligned.

## Discussion

Traditionally, genome sequence alignment was performed by giving a match score, mismatch score, and (open/extend) INDEL penalty, so that every base pair was given the same weight. We proposed the ZDP algorithm by giving each base pair a separate weight. Here, we applied ZDP sequence alignment algorithm on genome annotation lift over. Taking advantage of genetic features of genes and nearby sequences that can be grouped into a certain number of classifications, we accelerated the ZDP approach using single instruction, multiple data technology.

As a comparative genome annotation tool, the performance of GEAN highly relies on the reference genome annotation and genome assembly quality. The transformed genome annotation is expected to have similar or worse quality compared with the reference genome annotation. By checking the ORF state, GEAN decides whether to run the ZSDP algorithm. If there are ORF-shifts or premature stop-codon assembly errors, both the standard sequence alignment and ZSDP algorithm could not evaluate the ORF state correctly. We implemented a function to integrate lift over genome annotation, ZSDP, and other sources of genome annotation. Results produced by GEAN might be used as a very reliable information source for a standard gene structure combining implementation, e.g., EVidenceModeler (Haas et al., 2008).

Here, we tested our GEAN software using *Drosophila*, *Arabidopsis*, *Cardamine*, and maize. The inconsistent alignments affect more transcripts in a more diverse population. For individuals with high sequence similarity, GEAN has only a small advantage over using the standard sequence alignment gene structure lift over. In general, there is an incredible amount of variation in plant populations, for example, the divergence between two maize lines is approximately equivalent to the difference between humans and chimpanzees (Chen and Li, 2001). Future analyses utilizing GEAN can expand beyond plants to perform sensitive sequence alignments.

With decreasing costs of long-read sequencing technology, population genetics analyses are moving from short-read-based variant calling to long-read *de novo* genome assembly on a population scale. Lift over the high-quality reference genome annotation to the *de novo* assembly is becoming a standard analysis. Genome alignment approaches like GEAN can use the sequence context around an exon to correctly align the exon boundaries, so that it could align short CDS/exon where the accuracy of spliced aligners is limited (König et al., 2018), while spliced aligners could align genes with intron deletion or novel intron better.

As the earlier examples demonstrate, the capabilities of GEAN enable transforming the genome annotation of any two or collections of genomic sequences by using computer facilities that are widely available today. By refine standard sequence alignment and left alignment, we anticipate that GEAN will be applied to *de novo* genome assembly population genetics.

## Software

The gene structure annotation alignment is implemented in the GEAN software. Related functions, such as coordinate lift over any position, obtaining the pseudo-genome sequence, and checking the ORF states, are also implemented. Additionally, a function that lifts over all the reference annotation to other accessions is implemented.

GEAN is written in C++ and parallelized using the C++ Standard Library and freely available at https://github.com/baoxingsong/GEAN. A series of examples and tests is included in the distribution. By making the system open source, we hope to encourage others to expand and improve the code base.

## Data Availability Statement

Publicly available datasets were analyzed in this study. This data can be found here: https://github.com/baoxingsong/GEAN.

## Author Contributions

BS, QS, FW, and XG conceived this software. BS and QS structured the draft, and HW provided final editing. BS coordinated and drafted the manuscript and synthesized comments provided by all authors. All authors contributed critically important comments. BS implemented the software, and HP and FW performed testing and contributed source code. All authors read and approved the final manuscript.

## Funding

This work was supported by Natural Science Foundation of China grant #31900486 to BXS, Guizhou Provincial Science and Technology Foundation [(QKH Basic research) [2019]1298], Guizhou Provincial Education Department [QJH-KY-Z [2018]420], Qiannan Science and Technology Bureau [QNKHXKJSNX(2018)5], Research and Innovation Team of Qiannan Normal University for Nationalities (Qnsyk201605, QNYSKYTD2018011), and Scientific Research Project of Qiannan Normal University for Nationalities (qnsyrc201611, qnsyzw1809, QNSY2018ZJ006) grant to FW.

## Conflict of Interest

The authors declare that the research was conducted in the absence of any commercial or financial relationships that could be construed as a potential conflict of interest.
