# Supplementary material for: Complement Genome Annotation Lift Over Using a Weighted Sequence Alignment Strategy
Source: Front Genet. 2019 Nov 13;10:1046. doi: 10.3389/fgene.2019.01046 (PMC6902276; doi:10.3389/fgene.2019.01046)
Supplement: Supplementary file 1 [file DataSheet_1.docx]

**Supplementary Figure 1.** Directed acyclic graph of GO biological process terms that are enriched (dark orange) in a set of 5,122 genes showing different expression levels in at least 5 out of 697 accessions in which the RNA-seq reads mapping rate was greater than 90 using both Col-0 genome sequence and pseudo-genome as reference.

**
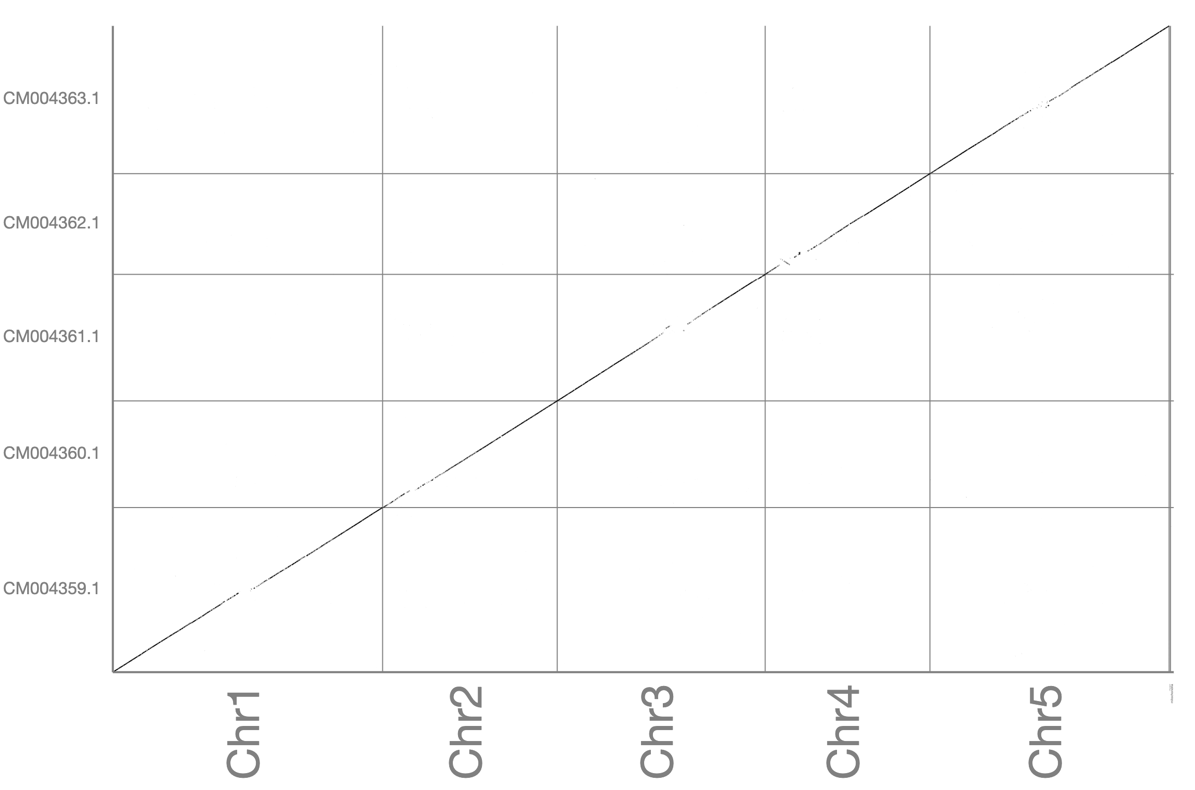
**

**Supplementary Figure 2.** The sequence alignment plot between *Arabidopsis thaliana* Col-0 and L*er*-0 generated using MUMmer4 + Assemblytics pipeline.
